# Bioinformatic analysis of chromatin organization and biased expression of duplicated genes between two poplars with a common whole-genome duplication

**DOI:** 10.1038/s41438-021-00494-2

**Published:** 2021-03-10

**Authors:** Le Zhang, Jingtian Zhao, Hao Bi, Xiangyu Yang, Zhiyang Zhang, Yutao Su, Zhenghao Li, Lei Zhang, Brian J. Sanderson, Jianquan Liu, Tao Ma

**Affiliations:** 1 College of Computer Science & Medical Big Data Center of Sichuan University & Key Laboratory of Bio-Resource and Eco-Environment of Ministry of Education & College of Life Sciences, Sichuan University, 610065 Chengdu, China; 2 Key Laboratory of Systems Biology, Hangzhou Institute for Advanced Study, University of Chinese Academy of Sciences, Chinese Academy of Sciences, 310024 Hangzhou, China; 3 Department of Biology, West Virginia University, Morgantown, WV 26506 USA; 4 State Key Laboratory of Grassland Agro-Ecosystem, Institute of Innovation Ecology & College of Life Sciences, Lanzhou University, 730000 Lanzhou, China

**Keywords:** Molecular ecology, Evolution, Chromatin

## Abstract

The nonrandom three-dimensional organization of chromatin plays an important role in the regulation of gene expression. However, it remains unclear whether this organization is conserved and whether it is involved in regulating gene expression during speciation after whole-genome duplication (WGD) in plants. In this study, high-resolution interaction maps were generated using high-throughput chromatin conformation capture (Hi-C) techniques for two poplar species, *Populus euphratica* and *Populus alba* var. *pyramidalis*, which diverged ~14 Mya after a common WGD. We examined the similarities and differences in the hierarchical chromatin organization between the two species, including A/B compartment regions and topologically associating domains (TADs), as well as in their DNA methylation and gene expression patterns. We found that chromatin status was strongly associated with epigenetic modifications and gene transcriptional activity, yet the conservation of hierarchical chromatin organization across the two species was low. The divergence of gene expression between WGD-derived paralogs was associated with the strength of chromatin interactions, and colocalized paralogs exhibited strong similarities in epigenetic modifications and expression levels. Thus, the spatial localization of duplicated genes is highly correlated with biased expression during the diploidization process. This study provides new insights into the evolution of chromatin organization and transcriptional regulation during the speciation process of poplars after WGD.

## Introduction

Chromatin is the main carrier of eukaryotic genetic information. Recent developments in chromatin conformation capture technologies (such as Hi-C) have improved our understanding of the nonrandom organization of chromatin and its important role in the regulation of gene expression^1–3^. There is growing evidence that most eukaryotic genomes are organized hierarchically^3–7^, including megabase-sized A/B compartments, topologically associating domains (TADs) from hundreds of kilobases to megabases in length, and smaller chromatin loops. These studies demonstrated correlations among chromatin interactions, epigenetic modifications, and transcriptional activity. However, because almost all of these studies have focused on single organisms, we lack a clear understanding of the evolutionary stability or lability of these hierarchically structured units of 3D organization of the genome^5^. The role of chromatin organization on interspecific variation in gene regulation, which is important in phenotypic and adaptive divergence between species, is just beginning to be studied^8–10^.

Our current understanding of the conservation of chromatin organization comes mainly from comparative studies in mammals, usually between distantly related species (such as humans and mice) or between primates^7,11,12^. Despite the distant evolutionary relationships among the studied mammals, there is remarkably high evolutionary conservation of chromatin organization among these species^8^. Further studies have shown that when there is divergence in chromosome conformation and the 3D localization of genes, there is typically a concomitant divergence in gene expression^5^.

Differential chromatin organization among species has not been widely investigated in plants. It is well known that angiosperms have undergone several rounds of whole-genome duplication (WGD) and subsequent gene loss and diploidization (genome fractionation), which is considered to be an important driver of the evolution of novel traits^13,14^. Previous studies have described chromatin organization in the model plant species *Arabidopsis thaliana*, as well as between distantly related crop species^15–23^. These studies have found that, with the exception of *A. thaliana* and *Arabidopsis lyrata*, the investigated plants exhibit many of the same features of chromatin organization found in animal species^24^. However, the relationship between genome organization and gene regulation during the process of genome fractionation remains elusive^21–23^. A recent study in *Brassica* species suggested that the spatial organization of WGD-derived paralogs is correlated with their biased retention and the eventual formation of subgenome dominance during the diploidization process after recent WGD^23^. However, the effects of chromatin organization on the transcriptional regulation of paralogs in plants that do not show subgenome dominance after WGD remain unknown. Further investigation into whether and how chromatin organization and expression of duplicated paralogs differ among closely related, uncultivated plant species may provide greater insight into the role of 3D genome structure in the diversification of plant species following WGD.

Poplar species (members of the genus *Populus*) are widely cultivated as a source of woody biomass, and due to the availability of a wide range of genomic resources, they are often used as a model tree species in molecular biology and genetics studies^25,26^. The genomes of all poplar species underwent a common ancient “Salicoid” WGD event, followed by diploidization, and maintained an extraordinarily stable karyotype with a basic haploid chromosome number of 19 (refs. ^27–31^). Previous studies revealed that the subgenomes of poplar do not show any signal of differential gene fractionation, but exhibit extensive divergence of expression between WGD-derived paralogs^32,33^. This provides an ideal system for studying the evolutionary dynamics of chromatin organization during speciation following a WGD and its possible effects on divergence in gene expression between species. In the current study, we combined Hi-C, DNA methylation, and gene expression data to examine the similarities and differences in hierarchical chromatin organization between two poplar species, *P euphratica* and *P. alba* var. *pyramidalis*, from two major clades of the genus that diverged ~14 million years ago and share a high degree of synteny^34–36^. We found that chromatin status was strongly associated with epigenetic modifications and gene transcriptional activity in both species, yet the chromatin organization showed surprisingly low conservation between the species. We also found that the divergence of gene expression between WGD-derived paralogs was associated with the strength of chromatin interaction. Colocalized paralogs exhibited great similarities in epigenetic modification and expression levels, suggesting that the spatial localization of duplicated genes was correlated with biased expression in the diploidization process. Overall, our results provide novel insights into the evolutionary lability of chromatin organization and transcriptional regulation during further speciation after a WGD.

## Results

### **A**n improved reference genome of *P. alba* var. *pyramidalis*

To identify the major structural variation between the genomes of these two species, we first produced a chromosome-level genome assembly of *P. alba* var. *pyramidalis* using single-molecule sequencing and chromosome conformation capture (Hi-C) technologies, and then performed comparative genomic analysis with a recently published genome assembly of *P. euphratica*^37^. The resulting assembly of *P. alba* var. *pyramidalis* consisted of 131 contigs spanning 408.08 Mb, 94.74% (386.61 Mb) of which were anchored onto 19 chromosomes (Supplementary Fig. S1 and Supplementary Tables S1–S3). A total of 40,215 protein-coding genes were identified in this assembly (Supplementary Table S4). The content of repetitive elements in the genome of *P. alba* var. *pyramidalis* (138.17 Mb, 33.86% of the genome) is 188.94 Mb less than that of *P. euphratica* (327.11 Mb, 56.95% of the genome), which contributes greatly to their differences in genome size (Supplementary Table S5).

### 3D organization of the poplar genomes

To characterize the spatial organization and evolution of poplar 3D genomes at a high resolution, we performed Hi-C experiments using HindIII for *P. euphratica* and *P. alba* var. *pyramidalis*, generating a total of 482.95 million sequencing read pairs. These data were mapped to their respective reference genome sequences. After stringent filtering, 81.72 and 94.61 million usable valid read pairs were obtained in *P. euphratica* and *P. alba* var. *pyramidalis*, respectively, and used for subsequent comparative 3D genome analysis (Supplementary Table S6). In addition, we profiled the DNA methylation and transcriptomes of the same tissue samples to provide a framework for understanding the relationships among epigenetic features and 3D chromatin architecture in poplar.

**Fig. 1:**
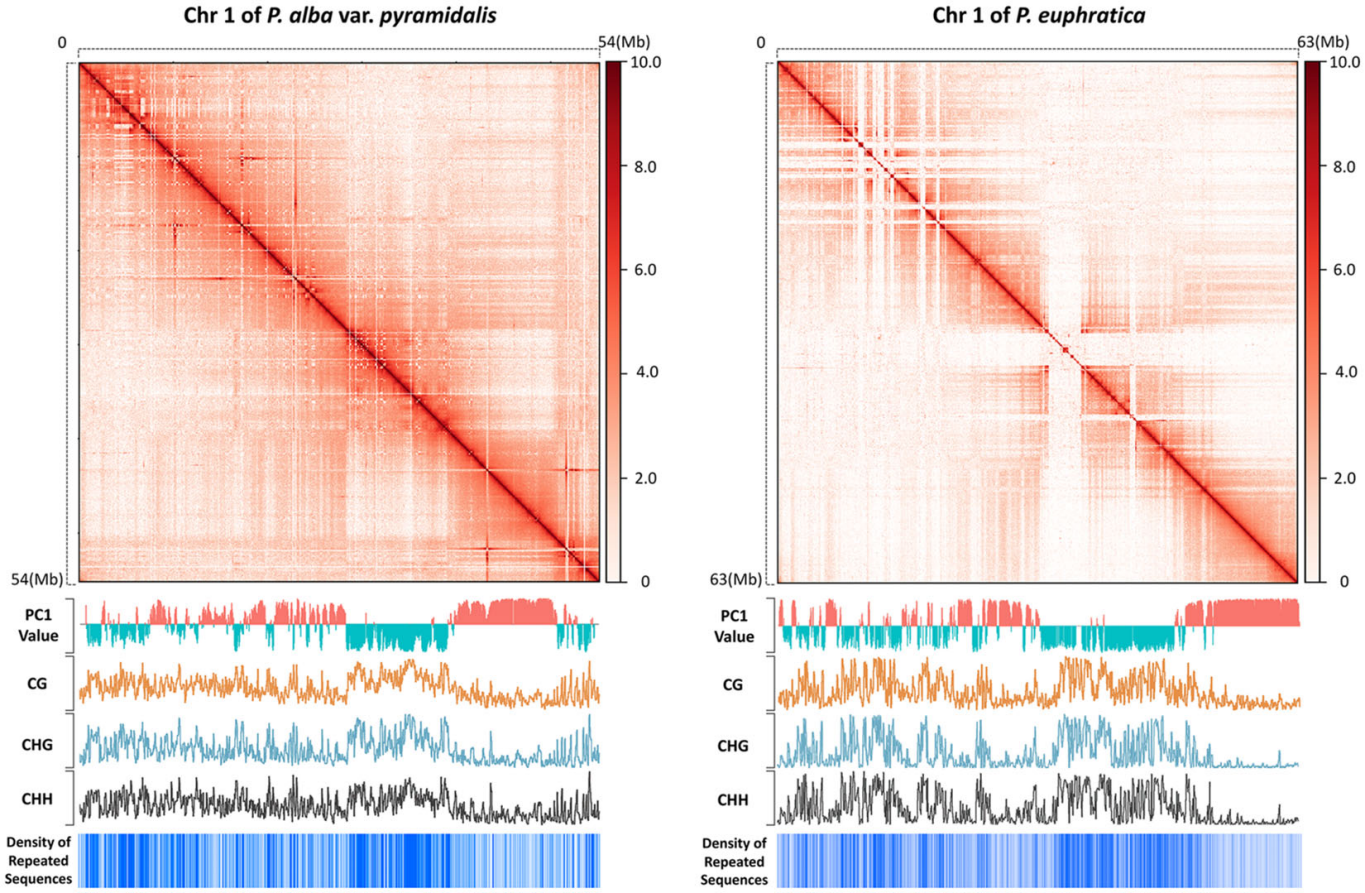
Hi-C heatmaps with compartment region analysis results at 50-kb resolution of *P. euphratica* chromosome 1 (left) and *P. alba* var. *pyramidalis* chromosome 1 (right). The heatmaps at the top are Hi-C contact maps at 50-kb resolution, which show global patterns of chromatin interaction in the chromosome. The chromosome is shown from top to bottom and left to right. The ICE-normalized interaction intensity is shown on the color scale on the right side of the heatmap. The track below the Hi-C heatmap shows the partition of A (red histogram, PC1 > 0) and B (green histogram, PC1 < 0) compartments as characterized by PCA. CG (yellow curve), CHG (gray curve), and CHH (red curve) methylation modification levels along the chromosome are plotted immediately below the compartment partition track. The heatmap at the bottom shows the density of repeat sequences along the chromosome

We first examined genome packing at the chromosomal level with a genome-wide Hi-C map at 50 kb binning resolution for *P. euphratica* and *P. alba* var. *pyramidalis*. As expected, the normalized Hi-C map from both species showed intense signals on the main diagonal (Fig. 1, and Supplementary Figs. S2 and S3) and a rapid decrease in the frequency of intrachromosomal interactions with increasing genomic distance, indicating frequent interactions between sequences close to each other in the linear genome (Supplementary Fig. S4). Strong intrachromosomal and interchromosomal interactions were also observed on the chromosome arms, implying the presence of chromosome territories in the nucleus, in which each chromosome occupies a limited, exclusive nuclear space^16,38^.

A common feature of all previous studies of chromatin organization is that regions of each chromosome are organized into “A” and “B” compartments, which correspond primarily to the euchromatic and heterochromatic regions, respectively^4–6^. To examine whether a similar compartment pattern also exists in poplar, we performed principal component analysis (PCA) on the genome-wide interaction matrix and categorized the genomic bins as A or B compartments according to the sign of the first principal component (PC1), with A compartments showing higher gene densities. The results indicated that ~56.72% of the *P. alba* var. *pyramidalis* genome belongs to A compartments, a significantly higher percentage than that in *P. euphratica* (53.09%; *P* = 2.173 × 10^−6^, two-sided Fisher’s exact test, *n* = 7743 in *P. alba* var. *pyramidalis* and *n* = 11,004 in *P. euphratica*; Supplementary Table S7). We found that interactions within each compartment were more frequent than those across compartments (Fig. 1 and Supplementary Fig. S3), and that the A compartment regions interacted more frequently with A compartments from different chromosomes than with B compartments in both poplar species (Supplementary Fig. S5). The genes in the A compartments displayed significantly higher transcription levels than those in the B compartments, while the B compartments exhibited significantly higher transposable element densities and higher levels of CG, CHG, and CHH methylation in both *P. alba* var. *pyramidalis* and *P. euphratica* (Fig. 1, and Supplementary Figs. S3 and S5). These results are consistent with patterns reported in other plant and animal species^6,16,39^.

**Fig. 2:**
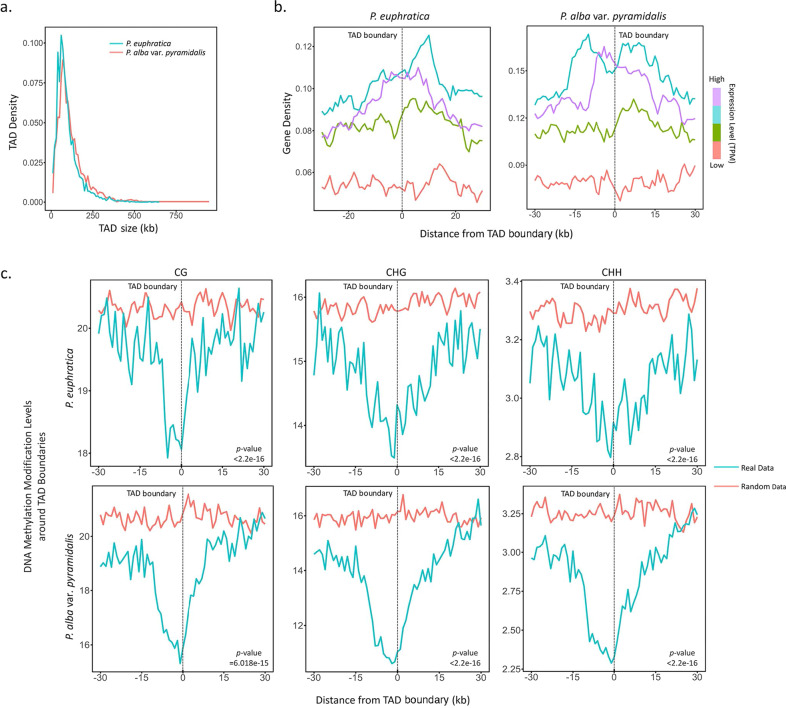
TAD profiles of *P. euphratica* and *P. alba* var. *pyramidalis*. **a** Size distribution of TADs of *P. euphratica* and *P. alba* var. *pyramidalis* at 10-kb resolution. **b** Density of genes with different expression levels (TPM) around TAD boundaries (±30 kb, window size = 1000 bp) in *P. euphratica* (left) and *P. alba* var. *pyramidalis* (right). Genes were divided into four groups based on the number of transcripts per kilobase of exon model per million mapped reads (TPM), and curves were color-coded based on these groups. **c** Distribution of DNA methylation levels (CG, CHG, and CHH) around TAD boundaries (±30 kb window size = 1000 bp) in *P. euphratica* and *P. alba* var. *pyramidalis*. The *y*-axis indicates the methylation level

A TAD is defined as a genomic region in which the interactions of the loci with each other tend to be more frequent than interactions with loci outside the region^7,40^. TADs are a common and prominent feature of the mammalian genome and have been shown to have profound effects on gene expression^4,5^. Recent studies have indicated that although few TADs have been identified in *Arabidopsis*^15,17^, they are ubiquitous in the genomes of rice, cotton, *Brassica*, and other crops^19–21,23^. To examine the existence of TADs in poplar, we employed the TopDom method^41^ on the 10-kb corrected interaction matrix of each individual chromosome. A total of 3175 and 4829 TADs with median sizes of 100 and 80 kb were identified in the genomes of *P. alba* var. *pyramidalis* and *P. euphratica*, and collectively covered ~97.34% and 86.28% of the genome lengths, respectively (Fig. 2a, and Supplementary Tables S8 and S9). As expected, these domains showed enriched interactions within the same domain, but less frequent interactions with loci located in adjacent domains (Supplementary Fig. S6). To understand the role of TADs in poplar genome organization, we further analyzed the available genomic features at the TAD boundaries. The results showed that protein-coding genes are more often localized at boundaries than in TAD regions. Prominent enrichment of highly expressed genes at the TAD boundaries was observed in both *P. alba* var. *pyramidalis* and *P. euphratica* (Fig. 2b). Consistent with these results, DNA methylation in the CG, CHG, and CHH contexts displayed an obvious decrease around the TAD boundaries (Fig. 2c). All of these results suggest that the active transcription and epigenetic modification might contribute to the formation of TADs in poplar, similar to findings in other plant species^19–21,23^.

### Comparison of 3D organization between the two poplar genomes

To study the evolutionary conservation of genome organization during the speciation of these two poplars, we conducted a whole-genome alignment and compared the distribution of compartments and TADs between the syntenic blocks. The results indicated extensive collinearity and similarity between these two genomes, with 298.66 Mb (73.19%) of *P. alba* var. *pyramidalis* sequences aligning with 299.69 Mb (52.17%) of *P. euphratica* sequences. Further analysis revealed that the majority (65.12%) of the unaligned regions resulted from the recent insertion of repetitive elements in the genome of *P. euphratica*. In total, we identified 19,235 large (>5 kb) structural variants ranging from 5 to 446 kb in length in the alignment of the two genomes, including 719 inversions, 476 translocations, and 7947 and 10,093 unique regions in *P. alba* var. *pyramidalis* and *P. euphratica*, respectively (Supplementary Tables S10 and S11).

**Fig. 3:**
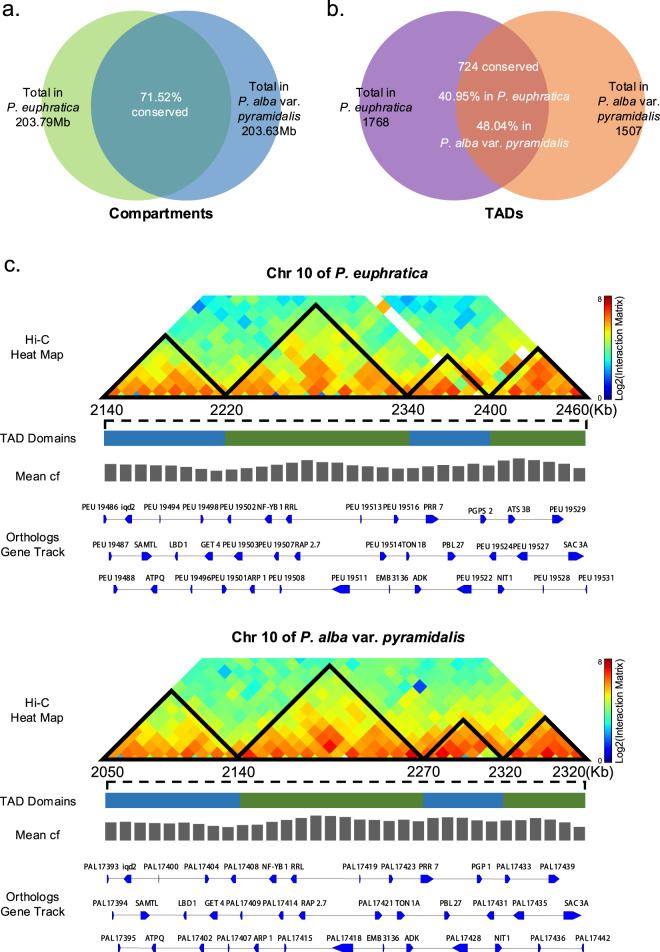
Evolutionary conservation of compartment status and TADs across *P. euphratica* and *P. alb*a var. *pyramidalis*. **a** Overlap of compartment status between syntenic regions in *P. euphratica* and *P. alba* var. *pyramidalis*. **b** Overlap of TADs between syntenic regions in *P. euphratica* and *P. alba* var. *pyramidalis*. **c** Example of conserved TAD structures across a syntenic region between *P. euphratica* and *P. alba* var. *pyramidalis*. The TADs are outlined by black triangles in the heatmaps, and the position of the TAD domains is indicated by alternating blue-green line segments. The mean cf value used to identify the domains is also shown. The orthology tracks of these conserved domains are shown at the bottom

**Fig. 4:**
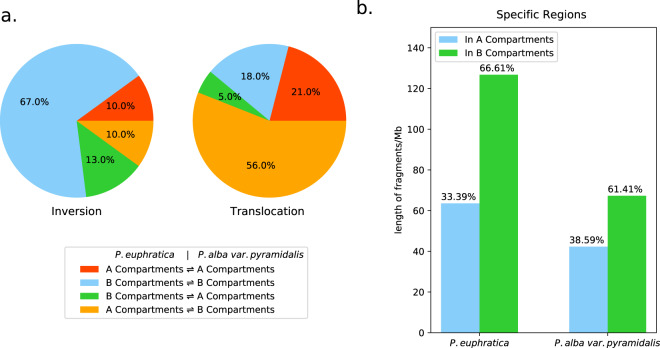
Relationship between structural variation and spatial organization of the genomes of *P. euphratica* and *P. alba* var. *pyramidalis*. **a** Analysis of compartment inversion (left) and translocation (right) across *P. euphratica* and *P. alba* var. *pyramidalis*. **b** Analysis of compartments of species-specific regions in *P. euphratica* (left) and *P. alba* var. *pyramidalis* (right)

To characterize the relationship between structural variation and spatial organization of the poplar genomes, we first analyzed the conservation of A/B compartments between *P. alba* var. *pyramidalis* and *P. euphratica*, using a 50-kb Hi-C matrix. The results showed that 71.52% (145.75 Mb in *P. euphratica* and 145.63 Mb in *P. alba* var. *pyramidalis*) of the total length of the syntenic regions have the same compartment status between the two species, while 43.68 and 43.71 Mb of the genomic regions exhibit A/B compartment switching in *P. alba* var. *pyramidalis* and *P. euphratica*, respectively (Fig. 3a). For the regions with structural variation, we found that 77% of the inversion events between the two genomes had no effects on their compartment status, while 61% of the translocation events occurred within the regions exhibiting compartment switching (Fig. 4a and Supplementary Table S10). Moreover, we also found that 38.59% and 33.39% of the nonsyntenic regions were identified as A compartments in *P. alba* var. *pyramidalis* and *P. euphratica*, respectively, indicating that the large-scale insertions and/or deletions are biased to occur at heterochromatic regions (Fig. 4b). We further assessed the conservation of genome organization at the TAD level by examining whether the orthologous genes within the same TAD in one species could still be located within the TAD in another species^19,21,23^. The results indicated that only 48.04% of TADs from *P. alba* var. *pyramidalis* and 40.95% from *P. euphratica* were substantially shared between the two species (Figs. 3b, c). Taken together, these results indicated that the 3D genome organization shows surprisingly low conservation across poplar species at both the compartmental and TAD levels.

### Relationship between chromatin interactions and expression divergence of WGD-derived paralogs

**Fig. 5:**
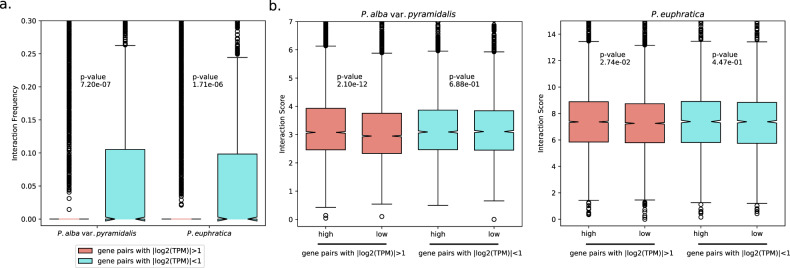
Comparison of interaction levels between WGD-derived paralogs with biased/similar expression in *P. euphratica* and *P. alba* var. *pyramidalis*. **a** The box plot shows that the interaction frequency of WGD-derived paralogs with biased (fold change > 2) and similar (fold change < 2) expression in *P. euphratica* and *P. alba* var. *pyramidalis*. Genes with low expression levels (TPM < 0.5) were discarded. The *y*-axis values indicate the ICE-normalized interaction frequency. **b** The box plots show the interaction score of WGD-derived paralogs with biased/similar expression in both *P. euphratica* and *P. alba* var. *pyramidalis*. The *y*-axis shows the interaction scores, which were calculated based on 10 kb O/E matrices

Poplar species have undergone a recent WGD event followed by diploidization, a process of genome fractionation that leads to functional and expression divergence of the duplicated gene pairs^27,28,33^. Although no biased gene loss or expression dominance was found between the two poplar subgenomes, there is evidence that nearly half of the WGD-derived paralogs have diverged in expression^32,33^. To explore the potential role of chromatin dynamics on the observed expression patterns of duplicated genes, we examined their differences in chromatin interaction patterns for both species. We first identified a total of 10,438 and 9754 paralogous gene pairs showing interchromosomal interactions in *P. euphratica* and *P. alba* var. *pyramidalis*, respectively. After correlating the frequency of chromatin interactions with their differences in expression, we found that gene pairs with biased expression (more than twofold differences in expression levels) interacted less frequently than gene pairs with similar expression levels in both species (*P* = 1.71 × 10^−6^ and 7.20 × 10^−7^ for *P. euphratica* and *P. alba* var. *pyramidalis*, respectively, Mann–Whitney *U* test; Fig. 5a). We also estimated the interaction score (the average of the distance-normalized interaction frequencies) for bins involved in the paralogous gene pairs and quantified their differences in interaction strength (Supplementary Fig. S7 and Supplementary Table S12)^3,23^. Our results showed that for gene pairs with biased expression, highly expressed gene copies have stronger interaction strengths than weakly expressed copies (*P* = 2.10 × 10^−12^ and 2.74 × 10^−2^ for *P. alba* var. *pyramidalis* and *P. euphratica*, respectively, Mann–Whitney *U* test), while no significant differences were observed for gene pairs with similar expression levels (Fig. 5b). We further investigated these phenomena at the level of high-order chromatin architecture and found that the gene pairs located in conserved TADs had similar expression levels (*P* = 2.68 × 10^−3^ and 7.86 × 10^−6^ for *P. euphratica* and *P. alba* var. *pyramidalis*, respectively, Mann–Whitney *U* test; Supplementary Fig. S8). Overall, our analyses indicate that the extensive expression divergence between WGD-derived paralogs in *Populus* is associated with the differences in their chromatin dynamics and 3D genome organization, and suggest that this organization may function as a key regulatory layer underlying expression divergence during diploidization.

**Fig. 6:**
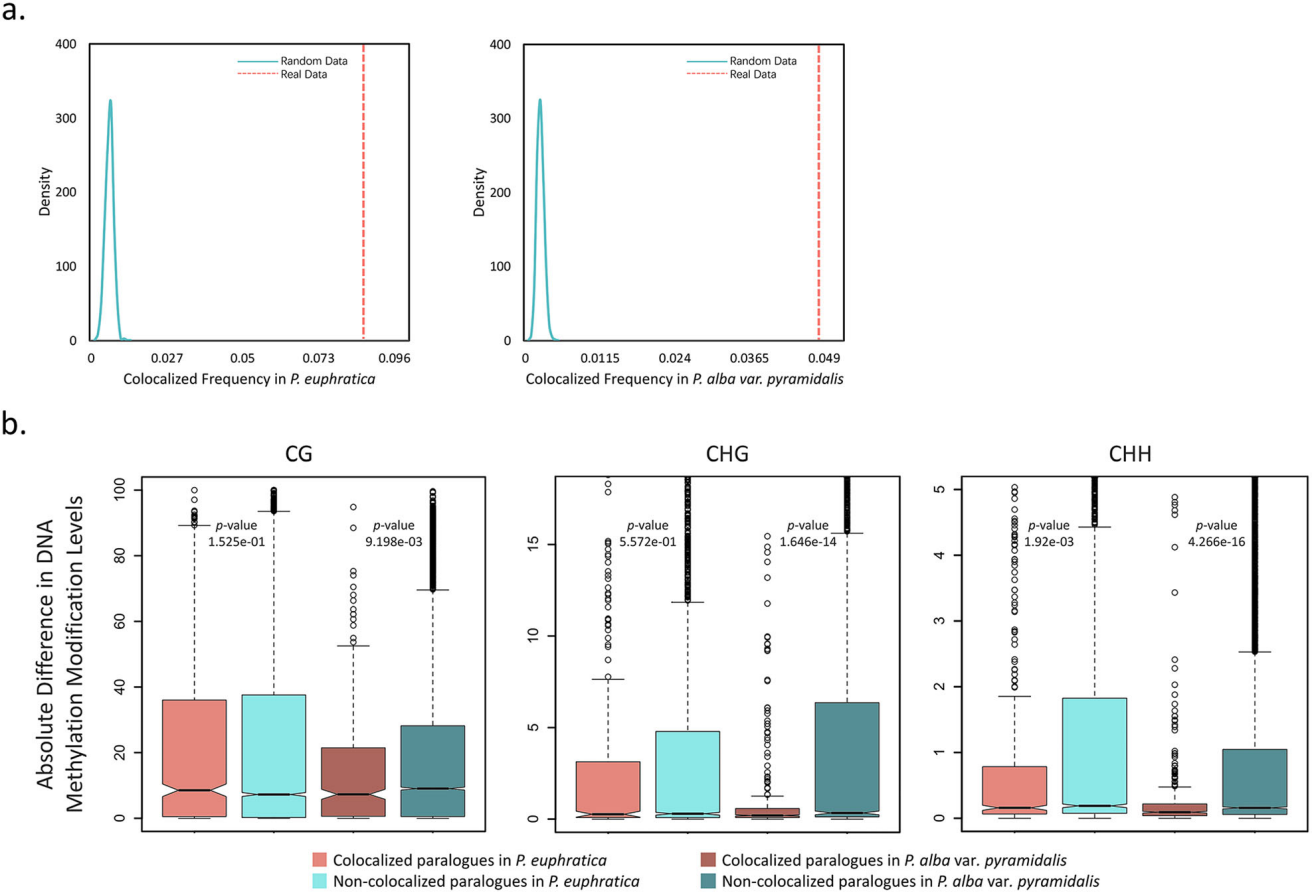
Colocalization frequency of interchromosomal paralogs retained after WGD and comparison of DNA methylation modification differences between colocalized and noncolocalized paralogs. **a** Colocalized interchromosomal paralog pairs in *P. euphratica* (left) and *P. alba* var. *pyramidalis* (right). The orange dashed lines indicate the observed colocalization frequencies for paralogs retained after WGD. The blue curves show the colocalization frequency distributions for 1000 randomizations of the same number of pairs as in the real data. **b** Box plots of the absolute difference in DNA methylation modification levels (CG, CHG, and CHH) between colocalized paralogs and between noncolocalized paralogs after WGD in *P. euphratica* and *P. alba* var. *pyramidalis*

In addition, we identified 849 and 454 spatially colocalized paralogs in *P. euphratica* and *P. alba* var. *pyramidalis*, respectively, which exhibited significantly stronger chromatin interactions than other gene pairs derived from WGD (false detection rate < 0.05). The number of colocalized paralogs was greater than that obtained from 1000 randomly selected samples, indicating that the spatial organization of the WGD-derived paralogs is not random and that they are more likely to be colocalized in both species (Fig. 6a). Further comparisons showed that these colocalized paralogs exhibited more similar DNA methylation patterns than noncolocalized gene pairs, especially in the “CHH” context (Fig. 6b). We finally examined the evolutionary conservation of the spatial colocalization, and the results showed that 198 of the colocalized gene pairs were orthologous between the two species. These overlapping genes accounted for 11.66% and 21.81% of the total colocalized paralogs in *P. euphratica* and *P. alba* var. *pyramidalis*, respectively, significantly higher proportions than expected by chance (3.89 and 7.38% at random, *P* < 2.2 × 10^−16^, two-sided Fisher’s exact test). These results highlight the conservation of colocalized paralogs and suggest that the spatial constraints of 3D genome organization might have functional significance under selective pressure.

## Discussion

The characteristics of genome 3D organization have been investigated in several model and crop plant species, and these studies identified prominent TADs as the common high-order structures of chromatin organization in most plants other than *Arabidopsis* species^15–23^. However, our knowledge of the evolutionary conservation of chromatin architecture, and its contribution to phenotypic and adaptative divergence between species is still in the early stages^24,42^. In this study, we present a comparative genome-wide analysis of chromatin interactions, and demonstrate the presence of A/B compartments and prominent TADs in both *P. euphratica* and *P. alba* var. *pyramidalis*. We found that the compartment status and TADs between these two poplar species showed substantially lower levels of conservation than those found among mammalian species^8^ and slightly lower levels of conservation than has been recently reported between closely related pairs of cultivated crop species^21,23^. We further show that compartment status and interaction strength are correlated with divergence in expression patterns among WGD-derived paralogous gene copies. Taken together, these results highlight the potential role of 3D genome organization in the evolutionary divergence of these species after a shared WGD.

Answers to the question of whether TADs are a common and conserved feature of plant genomes, as they are of mammalian genomes, have been rapidly shifting over the past decade as more species are studied. TADs were first reported in mice and humans, and subsequent studies found that the CTCF domains that contribute to the formation of these TADs show 50–75% conservation across 80 My (ref. ^8^). Early studies in *A. thaliana* and *A. lyrata* found that unlike in mammalian species, TADs were not a prominent feature of the *Arabidopsis* genome^15–17^. Subsequent studies have found TADs to be abundant in myriad crop species, leading some to conclude that the small genome/high gene density of *Arabidopsis* may preclude TAD formation^24^. A comparative analysis of five crop species found that there was little if any conservation of TADs among species^19^. However, a comparison of diploid and tetraploid cotton species (~1–2 My divergence) showed 70–80% conservation of TADs between the diploid species and the subgenomes of the tetraploid species^21^, and a similar comparison of *Brassica* spp. (~4 My divergence) showed 40–64% conservation of TADs^23^. Thus, the 41–48% conservation of TADs we observed between *P. euphratica* and *P. alba* var. *pyramidalis* (~14 My divergence) appears to be consistent with these more recent studies and highlights that even in uncultivated species, the conservation of genes that interact in TADs appears to break down over a relatively short evolutionary time scale in plants.

It is generally believed that changes in chromatin interactions play an important role in the divergence of gene expression^5^ and may even have been involved in biased gene retention during the diploidization of *Brassica*^23^. Consistent with this prediction, we observed stronger chromatin interactions for gene copies with higher expression levels between paralogs derived from WGD in both poplar species. We further identified a number of chromatin interactions between these paralogs and found that the frequency of interactions was negatively correlated with differences in their gene expression. This phenomenon was also confirmed for higher-order chromatin structures; that is, paralogs with the same compartment status or located in conserved TADs showed more similar expression levels. In addition, we found that the spatially colocalized paralogs exhibited strong similarities in epigenetic modifications and expression levels, indicating that chromatin interactions between paralogs may be an important regulatory layer in balancing gene expression and subgenome fractionation in poplar. Taken together, these results suggest a link between chromatin organization and biased expression of duplicated genes during the diploidization process in poplar. The conservation of these colocalized paralogs between the two species further indicates that the spatial localizations of these genes are maintained under evolutionary constraints. These results are consistent with those previously reported in *Brassica*, although unlike *Brassica*, poplar species do not exhibit broad patterns of biased gene loss or subgenome dominance. Rather, we find that differential regulation of retained paralogs in these poplars occurs through shifts in interaction strength and A/B compartment status.

In summary, our findings provide new insights into the structure and evolution of chromatin organization in poplars and highlight the potential importance of variation in chromatin structure in regulating paralogous copies via expression divergence during speciation after WGD. These results will accelerate our understanding of 3D genome evolution and its impact on transcriptional regulation in plants.

## Material and methods

### Plant material, Hi-C experiments, and sequencing

Two-year-old seedlings of *P. euphratica* and *P. alba* var. *pyramidalis* were planted in pots with loam soil, and grown in a greenhouse with a 16 h/8 h day/night photoperiod and 60% humidity at 25 °C. Nearly 2 g of fresh young leaves of each sample was ground to powder in liquid nitrogen for the Hi-C experiment. The Hi-C library was constructed following procedures described previously^37^, including chromatin extraction and digestion and DNA ligation, purification, and fragmentation. DNA libraries were constructed using an Illumina TruSeq DNA Sample Prep Kit and sequenced on an Illumina HiSeq X Ten system. The harvested material not subjected to a cross-linking reaction was used for RNA sequencing (RNA-seq) and whole-genome bisulfite sequencing (WGBS-seq) using strategies described previously^43^.

### Genome sequencing, assembly, and annotation of *P. alba* var. *pyramidalis*

Genomic DNA of *P. alba* var. *pyramidalis* was extracted using the CTAB (cetyl trimethylammonium bromide) method^44^. Then, 20-kb SMRTbell libraries were prepared according to the manufacturer’s protocol and sequenced on the PacBio Sequel platform (Pacific Biosciences, Menlo Park, CA, USA). Low-quality PacBio reads were removed, and the remaining subreads were base error corrected and assembled into contigs by FALCON v0.3.1 (ref. ^45^), using the parameters “pa_HPCdaligner_option = -v -B128 -t16 -e.70 -k16 -h300 -l3000 -w8 -s500 -H10000 -T8, ovlp_HPCdaligner_option = -v -B128 -t16 -k18 -h480 -e.96 -l2000 -w8 -s500 -T8, falcon_sense_option = –output_multi –min_idt 0.70 –min_cov 4 –max_n_read 200 –n_core 8”. Then, the base calling of contigs was improved by mapping the PacBio and Illumina reads to the preassembled contigs using Quiver^46^ and Pilon^47^ with default parameters. Finally, potential duplicate haplotypes were identified and removed from the assembly using the Purge Haplotigs^48^ pipeline. The construction of chromosome-level assemblies, genome annotation, and the identification of structural variants between the genomes of *P. euphratica* and *P. alba* var. *pyramidalis* were conducted, using procedures described previously^37^.

### Hi-C read mapping and normalization

Hi-C reads of *P. alba* var. *pyramidalis* and *P. euphratica* were aligned to the reference genome using Bowtie2 (v2.3.2)^49^, with default parameters. Each side of the paired end reads was mapped separately. Singleton reads, multimapped reads, and duplicated read pairs were removed by the quality control module of HiC-Pro (v2.10.0)^50^; therefore, only pairs for which both reads could be uniquely aligned were retained to identify valid interactions. Raw contact matrices were constructed with bin sizes of 10 and 50 kb and normalized using the ICE (iterative correction and eigenvector decomposition) method implemented in HiC-Pro (v2.10.0)^50^. Distance-normalized (observed/expected) matrices with 10-kb resolution were generated by a custom script for each chromosome of the two poplar species^6^. Heatmaps of the ICE and distance-normalized matrices were plotted using HiCPlotter (v0.6.6)^51^.

### Identification of genomic compartments and topologically associated domains

PCA implemented in HiTC software (v1.20.0)^52^ was applied to identify compartment regions on chromosomes of *P. alba* var. *pyramidalis* and *P. euphratica*. For each chromosome, genomic bins with a positive or negative value of the first eigenvector (PC1) were assigned to the A or B compartment, respectively. Regions with PC1 in the same direction with a greater number of genes corresponded to the A compartment, while regions with PC1 in the opposite direction belonged to the B compartment. TADs were detected based on 10 kb ICE-normalized matrices using TopDom software (v0.0.2)^41^, which has linear time complexity and depends on only a single, intuitive parameter. First, the *binSignal* value for each bin was generated by calculating the average contact frequency among pairs of upstream and downstream chromatin regions in a small window surrounding the bin. Then, the local minima in these *binSignal* values were designated as TAD boundaries. TADs that contained syntenic genes across *P. alba* var. *pyramidalis* and *P. euphratica* were compared to assess the evolutionary conservation of TADs. If the ratio of overlapping syntenic genes to the total number of syntenic genes in the compared TAD domains exceeded 70%, it was considered a conserved TAD. TADs that contained fewer than six genes were discarded.

### Identification of orthologs and WGD-derived paralogs

Protein sequences from *P. alba* var. *pyramidalis* and *P. euphratica* were all-vs-all aligned using blastp^53^, with the *E*-value set to 10^−5^. Then, MCScanX software^54^ was used to obtain the collinear relationships between these two species. To construct orthologous groups, the single-copy genes generated from OrthoMCL^55^ were used to identify a set of collinear fragments derived from speciation in combination with the results of MCScanX. To generate WGD-derived paralogs, intraspecific collinear fragments were selected, and the Ka/Ks ratios of these collinear fragments were calculated. The collinear fragments with Ks values in the range of 0.05–0.6 were chosen as the WGD-derived paralog fragments. The interchromosomal colocalized paralogs were then identified using the method described in previous studies, with a binomial distribution used to assign statistical significance and an FDR cutoff of 0.05 (ref. ^23^).

### WGBS-seq and data analysis

Genomic DNA was extracted for *P. alba* var. *pyramidalis* and *P. euphratica* using the CTAB method^44^. Three biological replicates from three individual seedlings were used to generate BS-seq libraries. The extracted DNA was mixed with the appropriate lambda DNA and fragmented by sonication to a mean size of 200–300 bp with a Covaris S220, followed by end-blunting, the addition of dA to the 3′-end, and adaptor ligation following the manufacturer’s protocol (Illumina). The procedure for bisulfite treatment of DNA and data analysis were described in our previous study^43^. Briefly, the potentially methylated cytosine sites were extracted using Bismark^56^ (version 0.16.3) software, with default parameters. Only sites that covered more than four mapped reads were retained.

### Gene expression analysis

To evaluate the gene expression of *P. euphratica* and *P. alba* var. *pyramidalis*, we mapped the RNA-seq data of leaf tissue from three replicates of *P. euphratica* and *P. alba* var. *pyramidalis*^57^ to their respective reference genome using HiSat2 (ref. ^58^), with default parameters. Next, the gene expression level of each gene (TPM value; transcript per million) was measured by StringTie^59^.

## Supplementary information

Supplementary Figures

Supplementary Tables

## Data Availability

All sequencing data generated for this study have been submitted to the National Genomics Data Center (NGDC; https://bigd.big.ac.cn/bioproject) under BioProject accession number PRJCA002423.
