# Supplementary material for: Bioinformatic analysis of chromatin organization and biased expression of duplicated genes between two poplars with a common whole-genome duplication
Source: Hortic Res. 2021 Mar 10;8:62. doi: 10.1038/s41438-021-00494-2 (PMC7943600; doi:10.1038/s41438-021-00494-2)
Supplement: Supplementary file 1 — Supplementary Figures [file 41438_2021_494_MOESM1_ESM.pdf]

Supplementary Figures

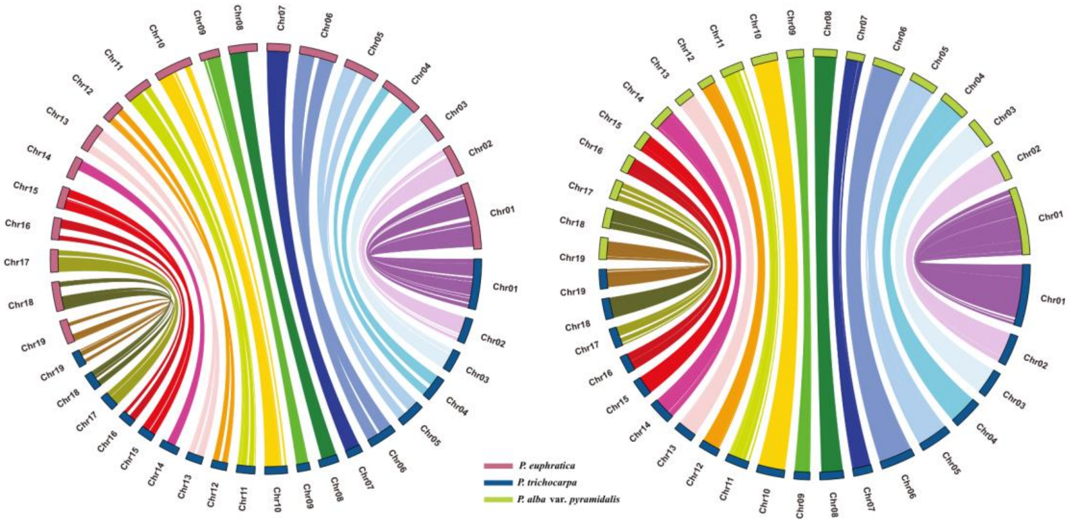

Fig. S1 Synteny relationships of *Populus euphratica*, *P. trichocarpa*, and *P. alba* var *pyramidalis* genomes.

a.

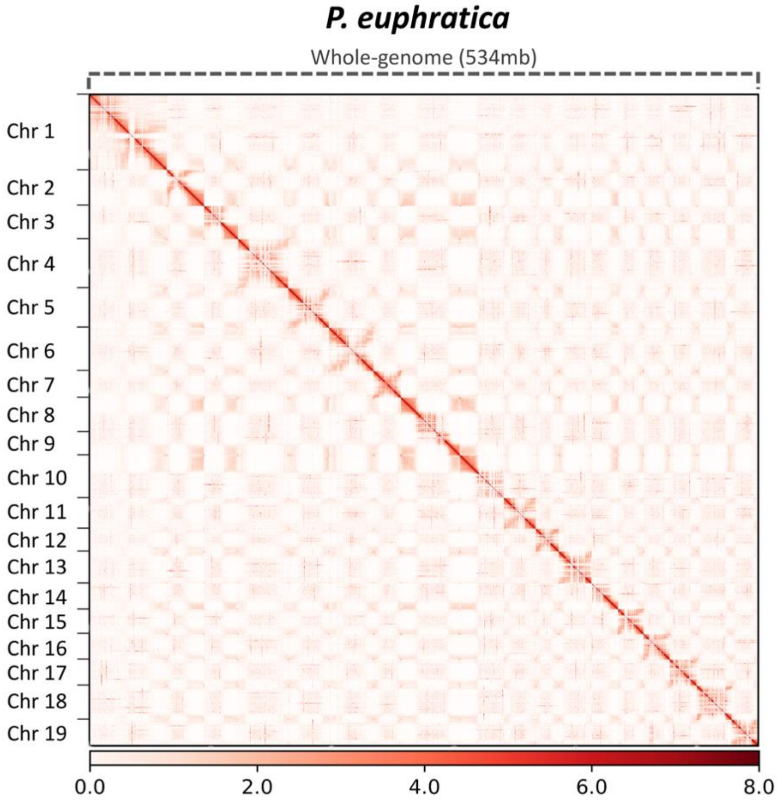

b.

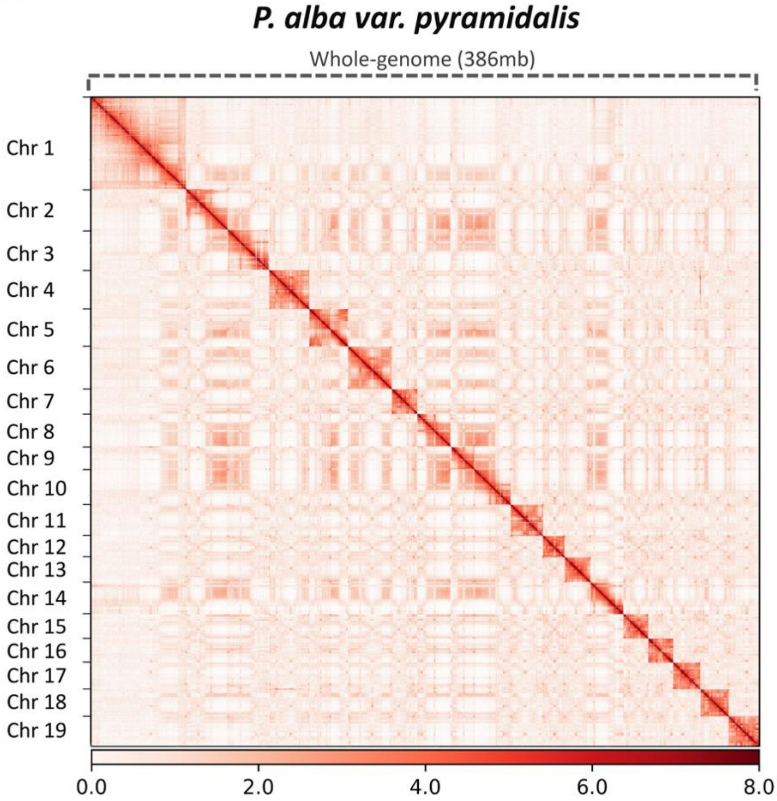

Fig. S2 Genome-wide Hi-C heat maps at 50kb resolution of *P. euphratica* (a) and *P. alba* var. *pyramidalis* (b). The heat map shows global patterns of chromatin interaction in the whole genome. 19 chromosomes are shown from left to right and top to bottom on the heatmap. The ICE-normalized interaction intensity is shown on the color scale on the bottom of heat map.

a.

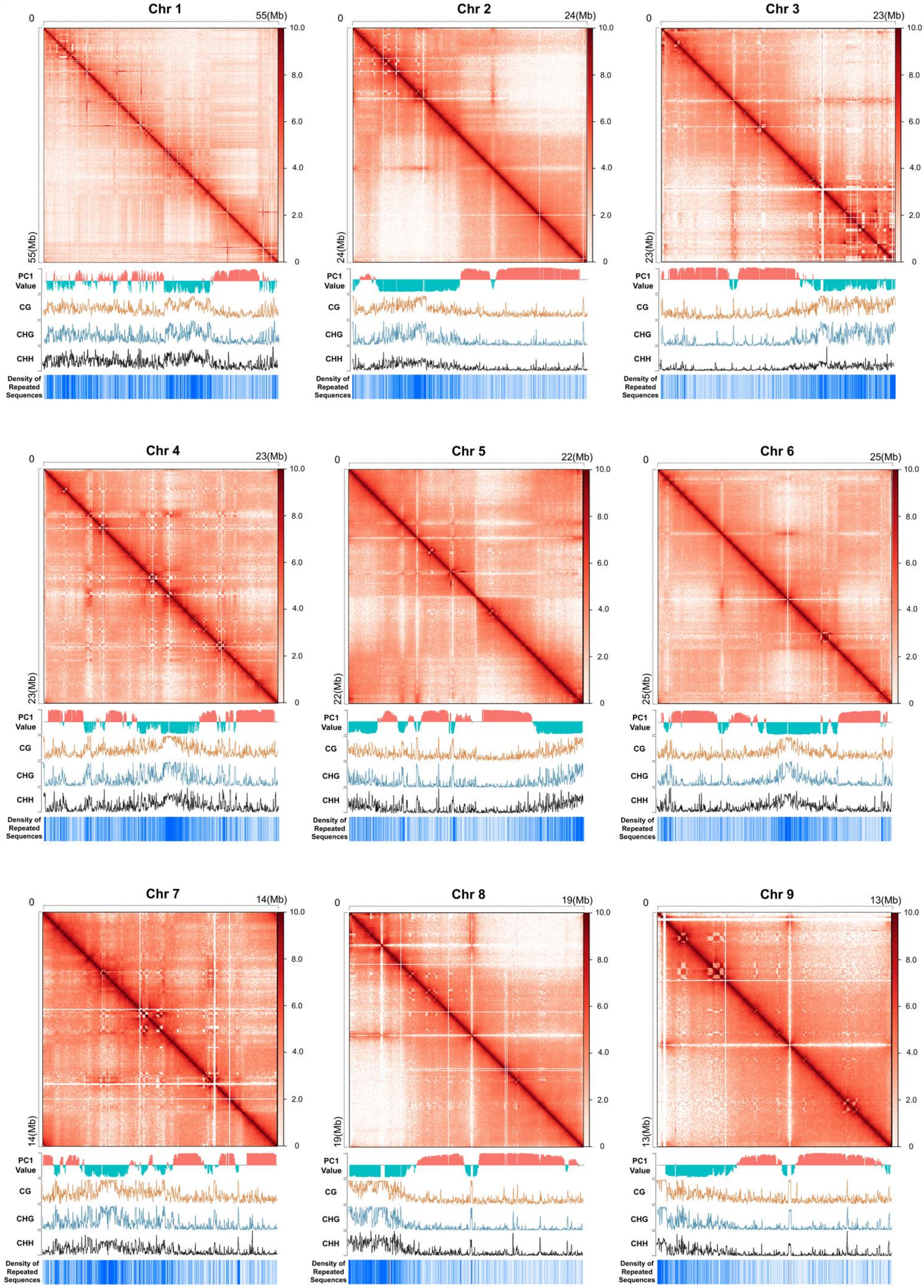

Chr 1
0
55(Mb)
55(Mb)
Chr 2
0
24(Mb)
24(Mb)
Chr 3
0
23(Mb)
23(Mb)
Chr 4
0
23(Mb)
23(Mb)
Chr 5
0
22(Mb)
22(Mb)
Chr 6
0
25(Mb)
25(Mb)
Chr 7
0
14(Mb)
14(Mb)
Chr 8
0
19(Mb)
19(Mb)
Chr 9
0
13(Mb)
13(Mb)
10.0
8.0
6.0
4.0
2.0
0
PC1 Value
CG
CHG
CHH
Density of Repeated Sequences

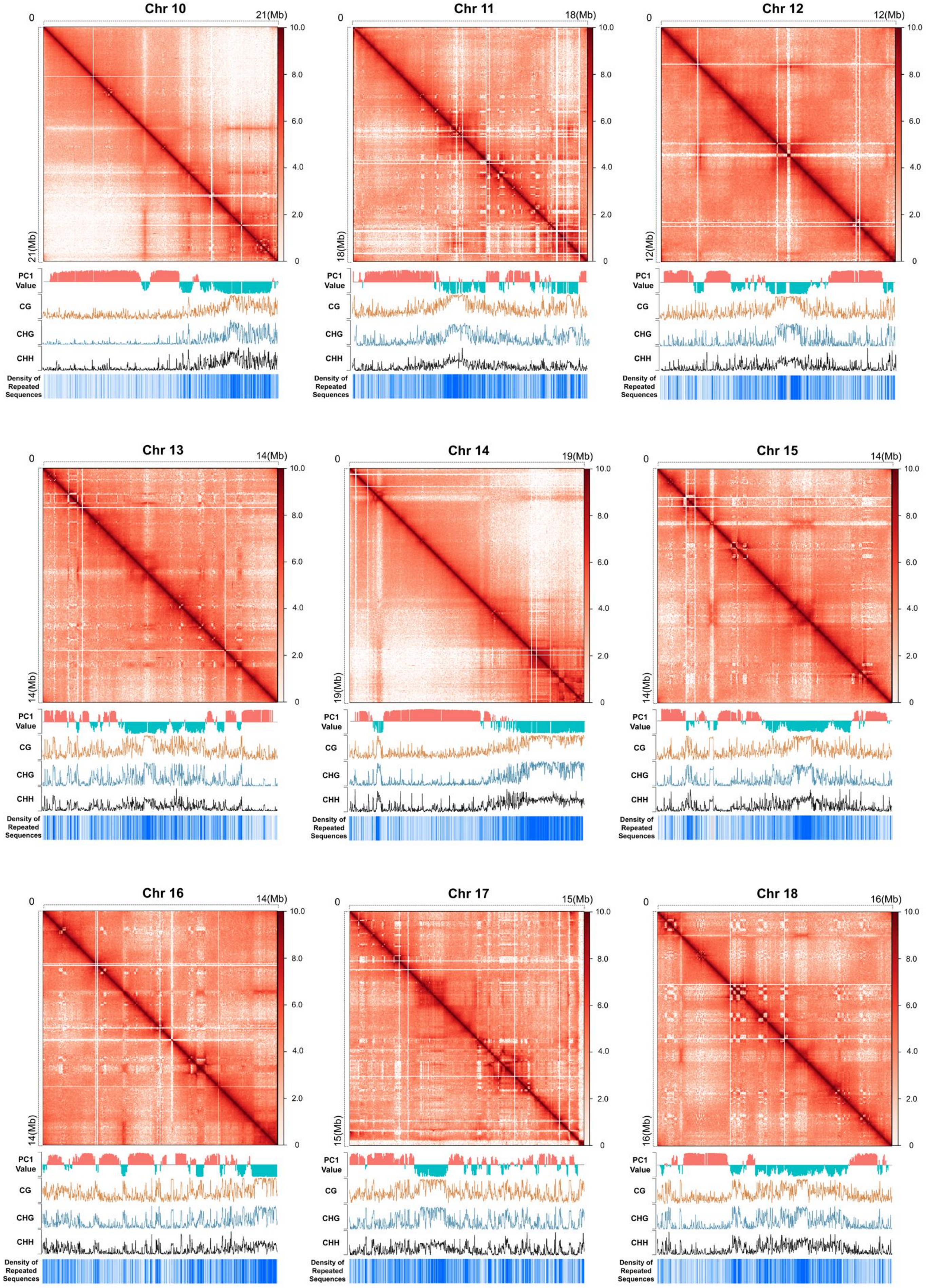

Chr 10
0
21(Mb)
21(Mb)
Chr 11
0
18(Mb)
18(Mb)
Chr 12
0
12(Mb)
12(Mb)
Chr 13
0
14(Mb)
14(Mb)
Chr 14
0
19(Mb)
19(Mb)
Chr 15
0
14(Mb)
14(Mb)
Chr 16
0
14(Mb)
14(Mb)
Chr 17
0
15(Mb)
15(Mb)
Chr 18
0
16(Mb)
16(Mb)
10.0
8.0
6.0
4.0
2.0
0
PC1 Value
CG
CHG
CHH
Density of Repeated Sequences

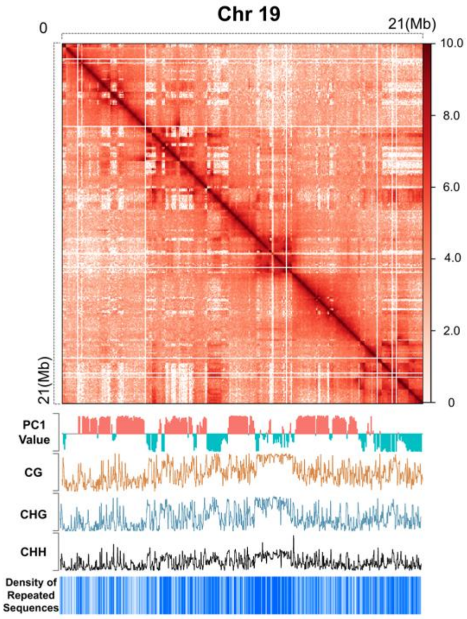

Chr 19
0
21(Mb)
21(Mb)
10.0
8.0
6.0
4.0
2.0
0
PC1
Value
CG
CHG
CHH
Density of
Repeated
Sequences

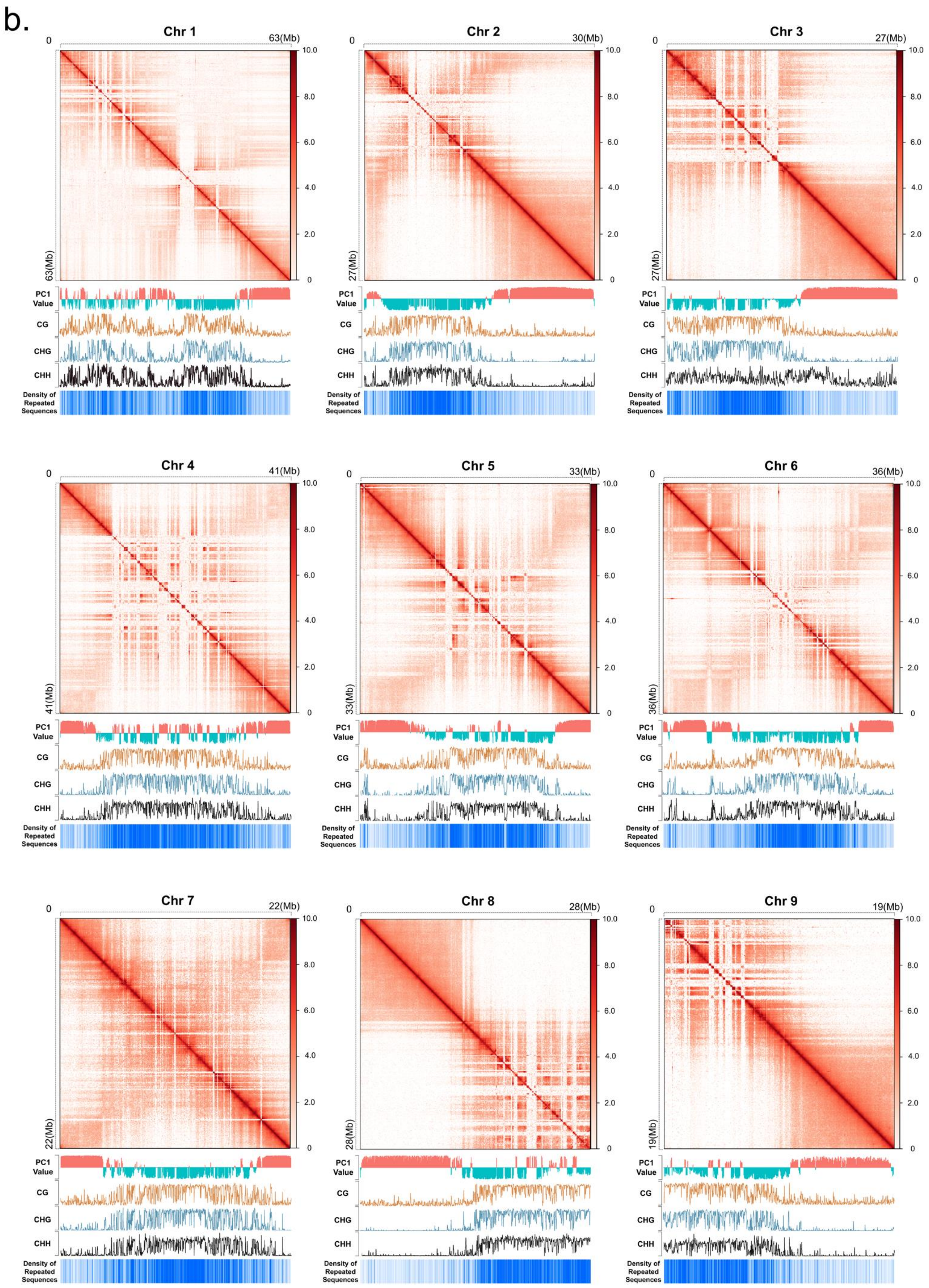

b.
Chr 1
0
63(Mb)
Chr 2
30(Mb)
27(Mb)
Chr 3
27(Mb)
Chr 4
41(Mb)
Chr 5
33(Mb)
Chr 6
36(Mb)
Chr 7
22(Mb)
Chr 8
28(Mb)
Chr 9
19(Mb)
10.0
8.0
6.0
4.0
2.0
PC1 Value
CG
CHG
CHH
Density of Repeated Sequences

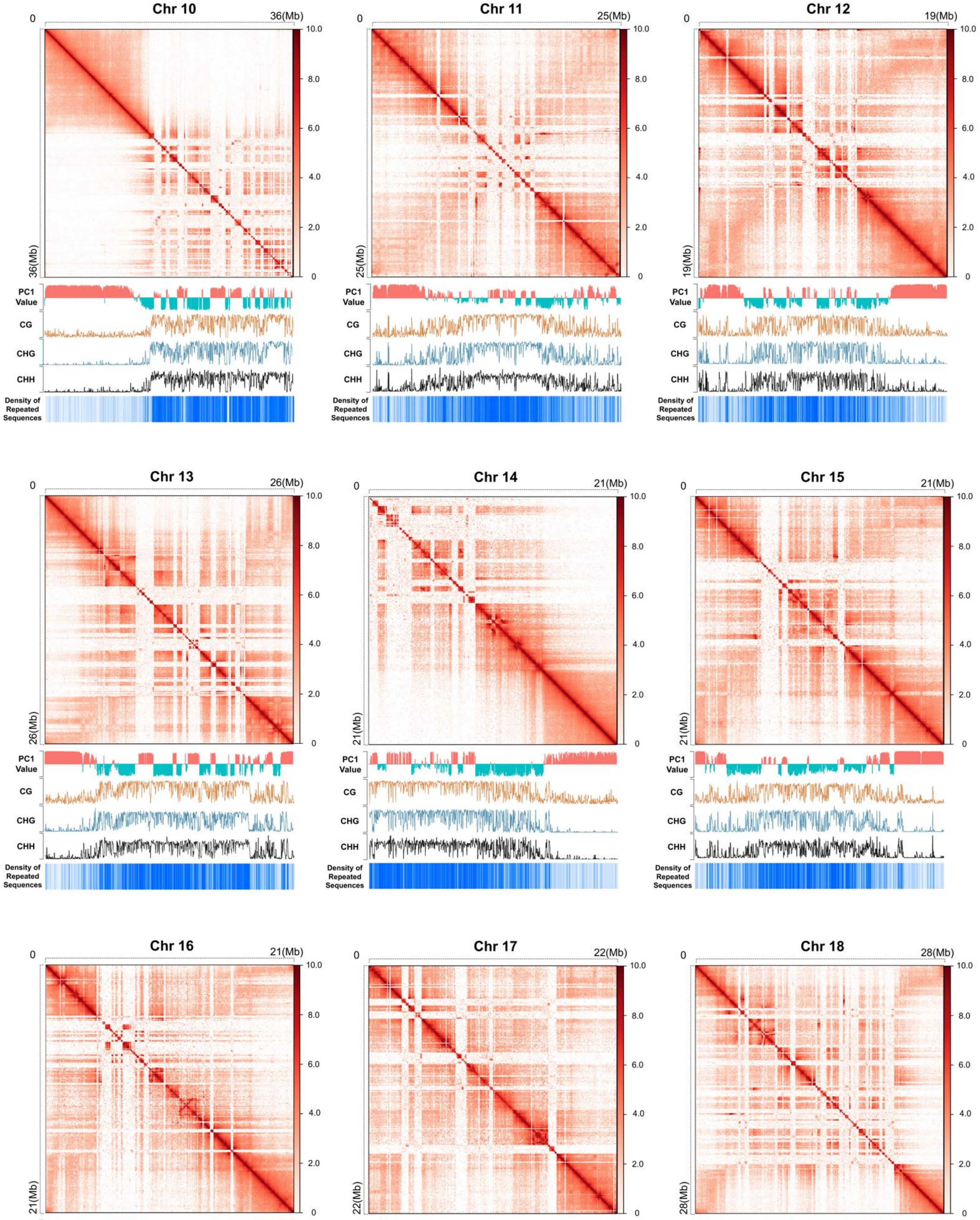

Chr 10
0
36(Mb)
36(Mb)
Chr 11
25(Mb)
25(Mb)
Chr 12
19(Mb)
19(Mb)
Chr 13
26(Mb)
26(Mb)
Chr 14
21(Mb)
21(Mb)
Chr 15
21(Mb)
21(Mb)
Chr 16
21(Mb)
21(Mb)
Chr 17
22(Mb)
22(Mb)
Chr 18
28(Mb)
28(Mb)
10.0
8.0
6.0
4.0
2.0
0
PC1 Value
CG
CHG
CHH
Density of Repeated Sequences

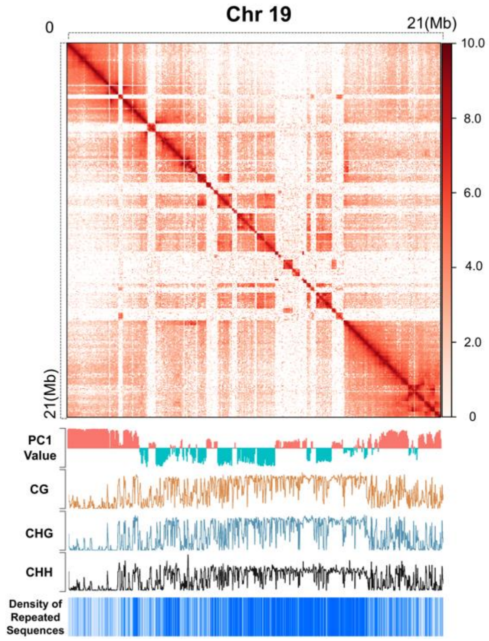

Fig. S3 Hi-C heatmaps with compartment region analysis results at 50-kb resolution of *P. alba* var. *pyramidalis* chromosome 1-19 (a) and *P. euphratica* chromosome 1-19 (b). The heat maps at the top are 50kb resolution Hi-C contact maps, show global patterns of chromatin interaction in the chromosome. Each chromosome is shown from top to bottom and left to right. The ICE-normalized interaction intensity is shown on the color scales on the right side of the heat maps. The tracks below the Hi-C heatmap show the partition of A (red histogram, PC1 > 0) and B (green histogram, PC1 < 0) compartments that characterized by PCA. CG (yellow curves), CHG (gray curves), and CHH (red curves) methylation modification levels along the chromosome are plotted right below the compartment partition tracks. The heat maps at bottom show the density of repeated sequences along each chromosome.

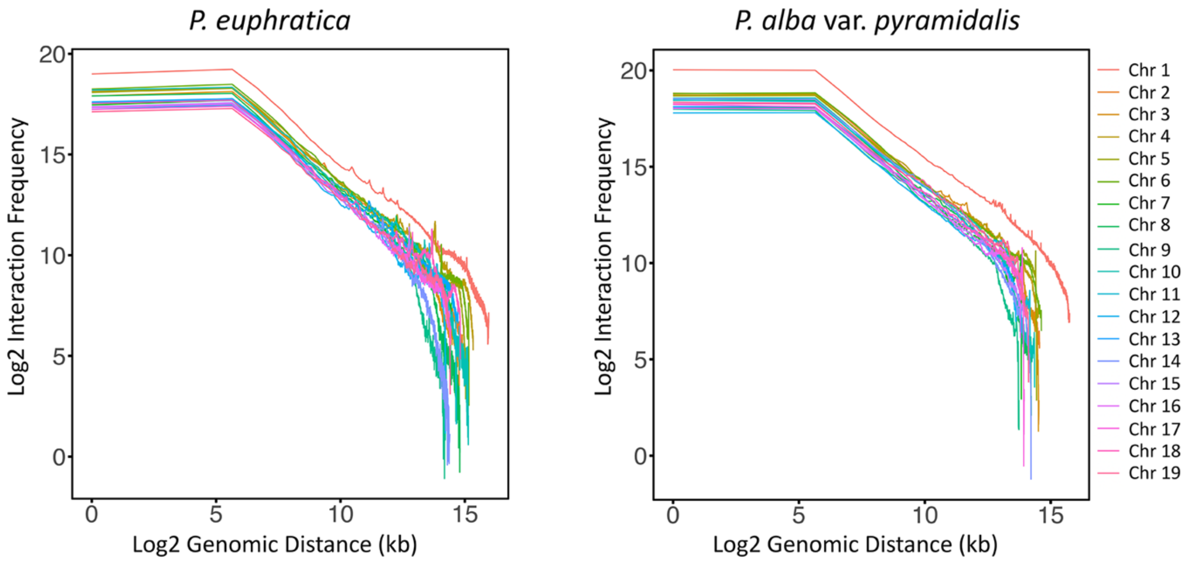

Fig. S4 Intra-chromosomal contact-distance analysis result of *P. euphratica* (Left) and *P. alba* var. *pyramidalis* (Right). Logarithm of ICE normalized contact frequency with logarithm of genomic distance was plotted separately for each chromosome at 50kb resolution.

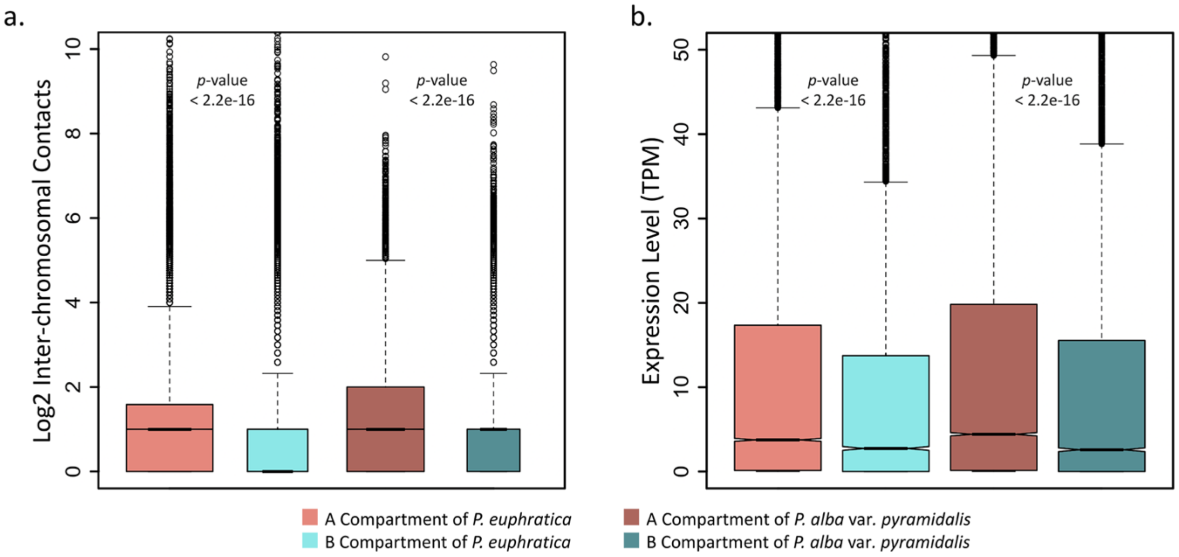

Fig. S5 Comparison of inter-chromosomal interactions and expression levels between compartment regions of *P. euphratica* and *P. alba* var. *pyramidalis*. a. The boxplot of inter-chromosomal contact between identical compartment regions of *P. euphratica* and *P. alba* var. *pyramidalis*. The width of the box indicates the number of bin pairs, and a wider box means more times of non-zero inter-chromosomal contact have occurred. **b.** Expression levels of different compartment regions in *P. euphratica* and *P. alba* var. *pyramidalis*.

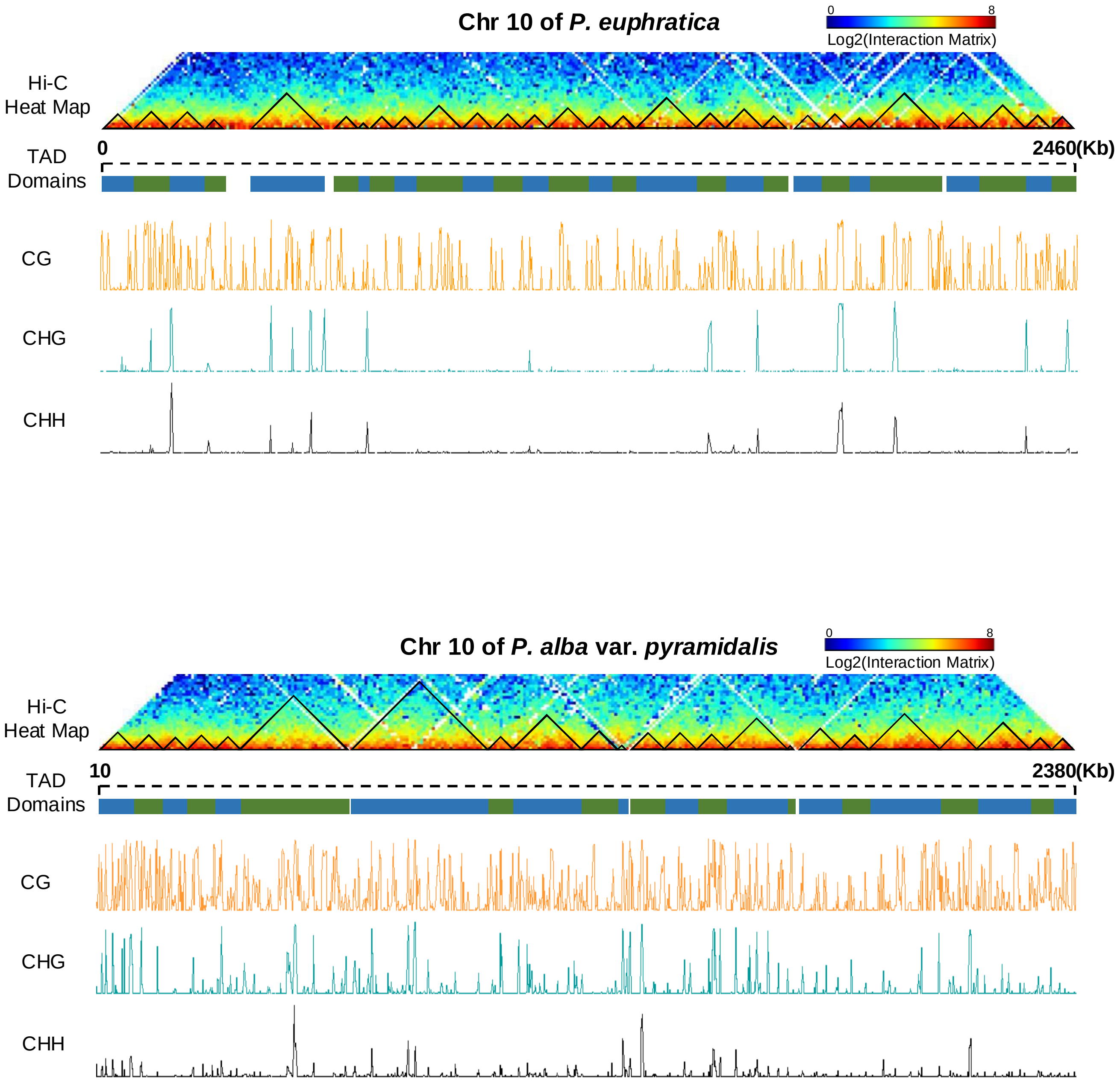

Fig. S6. Example of TAD structures over a syntenic region of chromosome 10 between *P. euphratica* and *P. alba* var. *pyramidalis*. The TADs are outlined by black triangles in the heatmaps, and the position of the TAD domains is indicated by alternating blue-green line segments.

a.

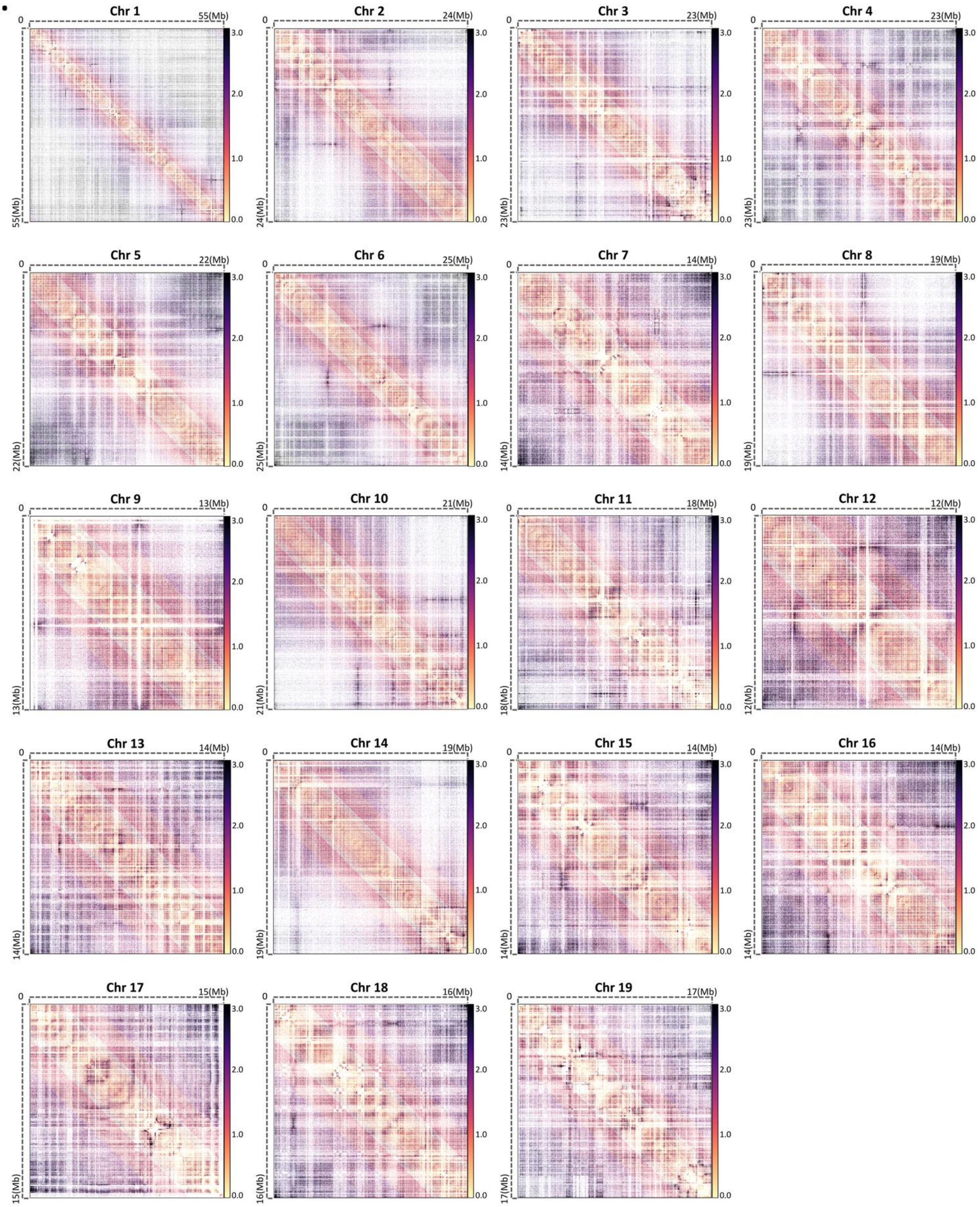

Chr 1
Chr 2
Chr 3
Chr 4
Chr 5
Chr 6
Chr 7
Chr 8
Chr 9
Chr 10
Chr 11
Chr 12
Chr 13
Chr 14
Chr 15
Chr 16
Chr 17
Chr 18
Chr 19

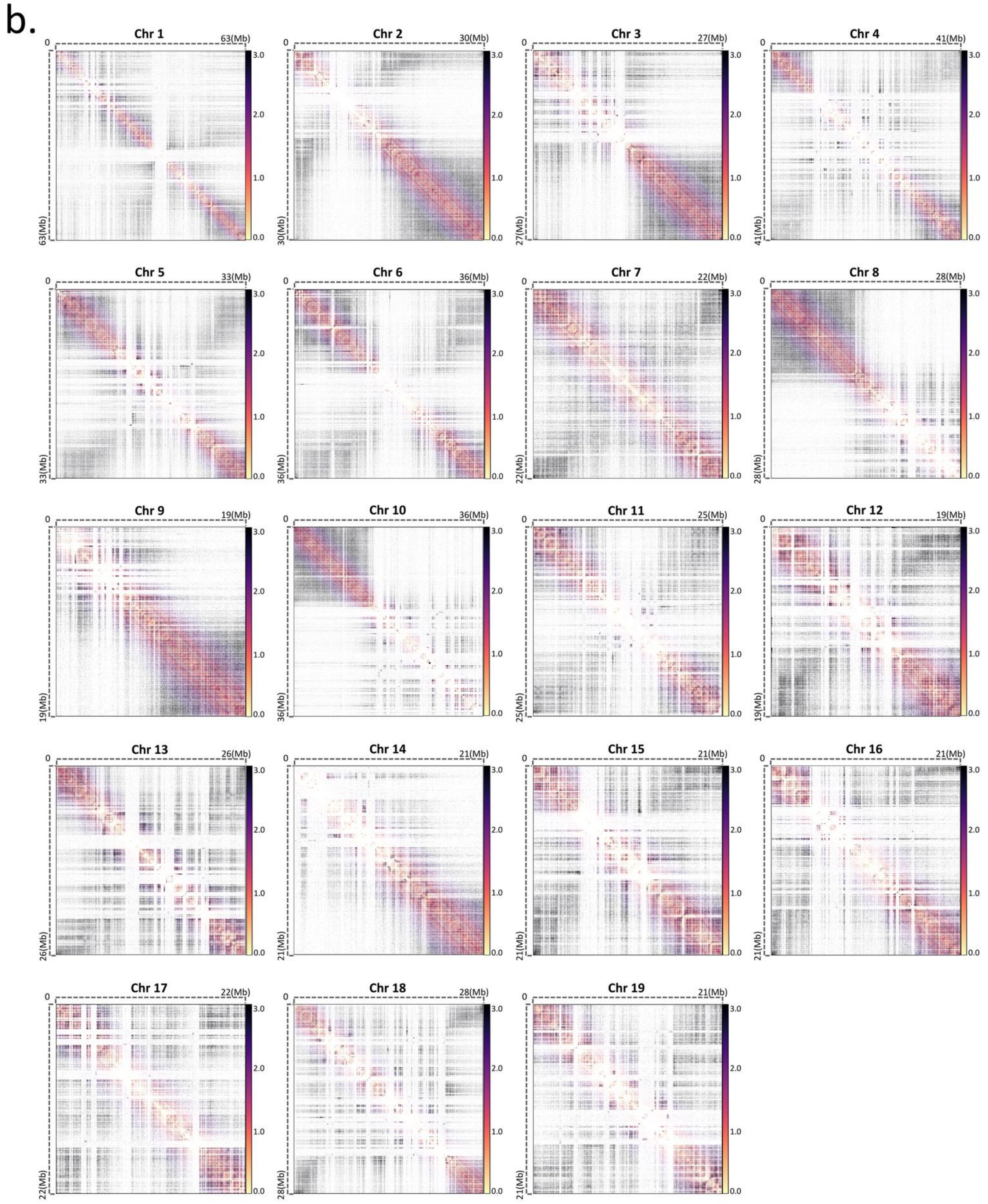

Fig. S7 Observed/Expected Heatmaps of each chromosomes of *P. alba* var. *pyramidalis* (a.) and *P. euphratica* (b.). The heat maps were generated from distance normalized (Observed/Expected) matrices at 50kb resolution. Each chromosome is shown from top to bottom and left to right. The distance-normalized interaction intensity is shown on the color scale on the right side of each heatmap.

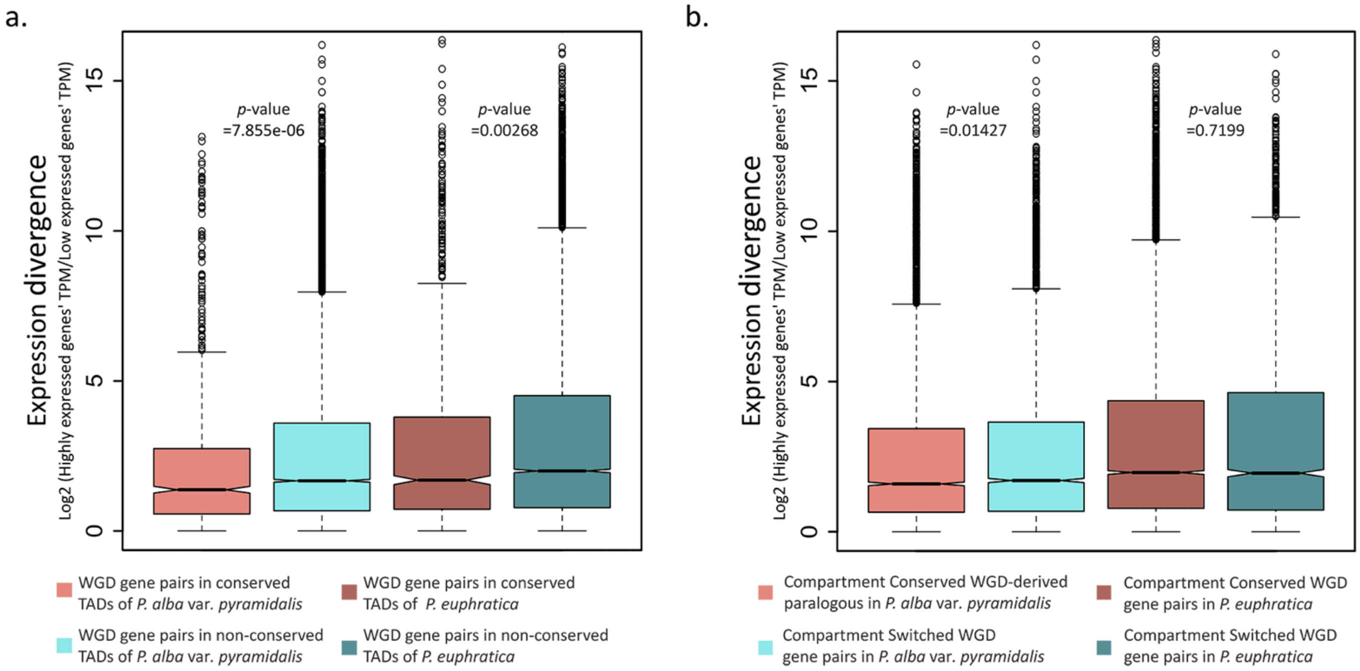

Fig. S8 Comparison of expression divergence between WGD-derived paralogous of *P. euphratica* and *P. alba* var. *pyramidalis*. a. Boxplot of expression divergence between WGD-derived paralogous in conserved/non-conserved TADs of *P. euphratica* and *P. alba* var. *pyramidalis*. **b.** Boxplot of expression divergence between compartment conserved/switched WGD-derived paralogous *P. euphratica* and *P. alba* var. *pyramidalis*.

Supplementary Tables

Supplementary Tables are listed in a separate .xlsx file. The titles of the supplementary tables are listed below.

Table S1. Summary for the chromosomal assembly of *P. alba* var. *pyramidalis* by Hi-C data.

Table S2 Comparison of the BUSCO analysis for genome assembly and gene annotation of *P. euphratica* (Peu) and *P. alba* var. *pyramidalis* (Pal).

Table S3 Evaluation of gene space completeness for the *P. euphratica* and *P. alba* var. *pyramidalis* genomes.

Table S4. Functional annotation of predicted genes for *P. alba* var. *pyramidalis.*

Table S5. Classification of repetitive elements in *P. alba* var. *pyramidalis* and *P. euphratica* genome.

Table S6. Hi-C Information of *P. euphratica* and *P. alba* var. *pyramidalis.*

Table S7. Compartment Regions list of *P. euphratica* and *P. alba* var. *pyramidalis.*

Table S8 Detection of TAD domains using different binning resolutions in *P. alba* var. *pyramidalis* and *P. euphratica*.

Table S9. TAD domains list of *P. euphratica* and *P. alba* var. *pyramidalis.*

Table S10. Characterization of chromosome inversions and translocations (with compartment statues) between *P. alba* var. *pyramidalis* and *P. euphratica.*

Table S11. Characterization of chromosome specific regions (with compartment statues) in *P. alba* var. *pyramidalis* and *P. euphratica.*

Table S12. Expression level and interaction score of WGD-derived paralogous in *P. euphratica* and *P. alba* var. *pyramidalis.*
